# Ultrasomics prediction for cytokeratin 19 expression in hepatocellular carcinoma: A multicenter study

**DOI:** 10.3389/fonc.2022.994456

**Published:** 2022-09-02

**Authors:** Linlin Zhang, Qinghua Qi, Qian Li, Shanshan Ren, Shunhua Liu, Bing Mao, Xin Li, Yuejin Wu, Lanling Yang, Luwen Liu, Yaqiong Li, Shaobo Duan, Lianzhong Zhang

**Affiliations:** ^1^ Department of Ultrasound, Henan University People’s Hospital, Henan Provincial People’s Hospital, Zhengzhou University People’s Hospital, Zhengzhou, China; ^2^ Henan Engineering Technology Research Center of Ultrasonic Molecular Imaging and Nanotechnology, Henan Provincial People's Hospital, Zhengzhou, China; ^3^ Department of Ultrasound, First Affiliated Hospital of Zhengzhou University, Zhengzhou, China; ^4^ Department of Ultrasound, Henan Provincial Cancer Hospital, Zhengzhou, China; ^5^ Department of Health Management, Henan University People’s Hospital, Henan Provincial People’s Hospital, Zhengzhou University People’s Hospital, Zhengzhou, China

**Keywords:** hepatocellular carcinoma, machine learning, radiomics, cytokeratin 19 (CK19), ultrasonography

## Abstract

**Objective:**

The purpose of this study was to investigate the preoperative prediction of Cytokeratin (CK) 19 expression in patients with hepatocellular carcinoma (HCC) by machine learning-based ultrasomics.

**Methods:**

We retrospectively analyzed 214 patients with pathologically confirmed HCC who received CK19 immunohistochemical staining. Through random stratified sampling (ratio, 8:2), patients from institutions I and II were divided into training dataset (n = 143) and test dataset (n = 36), and patients from institution III served as external validation dataset (n = 35). All gray-scale ultrasound images were preprocessed, and then the regions of interest were then manually segmented by two sonographers. A total of 1409 ultrasomics features were extracted from the original and derived images. Next, the intraclass correlation coefficient, variance threshold, mutual information, and embedded method were applied to feature dimension reduction. Finally, the clinical model, ultrasonics model, and combined model were constructed by eXtreme Gradient Boosting algorithm. Model performance was assessed by area under the receiver operating characteristic curve (AUC), sensitivity, specificity, and accuracy.

**Results:**

A total of 12 ultrasomics signatures were used to construct the ultrasomics models. In addition, 21 clinical features were used to construct the clinical model, including gender, age, Child-Pugh classification, hepatitis B surface antigen/hepatitis C virus antibody (positive/negative), cirrhosis (yes/no), splenomegaly (yes/no), tumor location, tumor maximum diameter, tumor number, alpha-fetoprotein, alanine aminotransferase, aspartate aminotransferase, alkaline phosphatase, glutamyl-transpeptidase, albumin, total bilirubin, conjugated bilirubin, creatinine, prothrombin time, fibrinogen, and international normalized ratio. The AUC of the ultrasomics model was 0.789 (0.621 – 0.907) and 0.787 (0.616 – 0.907) in the test and validation datasets, respectively. However, the performance of the combined model covering clinical features and ultrasomics signatures improved significantly. Additionally, the AUC (95% CI), sensitivity, specificity, and accuracy were 0.867 (0.712 – 0.957), 0.750, 0.875, 0.861, and 0.862 (0.703 – 0.955), 0.833, 0.862, and 0.857 in the test dataset and external validation dataset, respectively.

**Conclusion:**

Ultrasomics signatures could be used to predict the expression of CK19 in HCC patients. The combination of clinical features and ultrasomics signatures showed excellent effects, which significantly improved prediction accuracy and robustness.

## Introduction

Hepatocellular carcinoma (HCC) is the leading primary liver cancer, which is one of the major global health challenges (1). In 2020, liver cancer ranked sixth and third in incidence rate and mortality among all malignant tumors in the world, and there were approximately 905,000 new cases and 830,000 deaths (2). With a 5-year survival rate of 18%, liver cancer has become the second most fatal tumor, just secondary to pancreatic cancer (3). Although many treatment strategies are available in clinical practice, the recurrence rate of HCC remains high, and the prognosis is generally poor (4–6). Accumulating evidence suggests that HCC is a heterogeneous tumor with a multimolecular phenotype (7, 8), and that inter- and intratumoral heterogeneity is often highly resistant to clinical interventions, leading to treatment failure (9, 10). The key factors associated with the prognosis of HCC include microvascular invasion, tumor grade, Ki67 expression, etc. (11–13). Compared with these factors, CK19 is not only a prognostic marker of HCC (14), but also a stemness-related marker (15). Tumor hepatocytes are capable of self-renewal, differentiation and proliferation, with stronger tumorigenicity and chemoresistance (16, 17). Transarterial chemoembolization and systemic chemotherapy play an important role in the treatment of HCC (4, 6). However, CK19-positive HCC patients are more likely to develop resistance to chemotherapeutic drugs, resulting in treatment failure (18). Therefore, one manifestation of HCC heterogeneity is the expression of Cytokeratin (CK) 19.

Cytokeratins are important structural components in the epithelial cell skeleton (19). In adult liver, CK8 and CK18 are expressed in mature hepatocytes, while CK7 and CK19 are expressed in cholangiocytes and hepatic progenitor cells (20). CK19 has been shown to be expressed in 4-28% of HCC patients (21–23). *In vitro* studies have confirmed that CK19-positive HCC cells are closely related to invasiveness, epithelial-mesenchymal transition, and angiogenesis (23, 24). Compared with CK19-negative HCC patients, CK19-positive patients have a poorer prognosis, their clinical manifestations are not only more prone to resistance to chemotherapeutic drugs, but also have a higher incidence of extrahepatic metastasis and vascular invasion (14, 18, 23). Due to its high invasiveness, this molecular subtype has been considered as a new subtype of HCC (16, 25). It has been found that some liver transplant patients without CK19 expression and CK19-related gene expression have a good prognosis, even if they do not meet the Milan criteria (18, 24). It has been suggested that preoperative assessment of CK19 expression may help to determine judge whether patients beyond the Milan criteria meet the condition of liver transplantation, potentially expanding the criteria for liver transplantation (26). Therefore, preoperative assessment of CK19 expression in HCC patients is critical for the effective development of individualized treatment strategies.

Immunohistochemical analysis of biopsy tissues is a reliable method for the clinical preoperative assessment of CK19 expression in HCC patients (27). However, tissue biopsy is expensive, and the invasive procedure may bring a series of complications, such as intra-abdominal or subcapsular bleeding, needle-path metastasis and intra-abdominal metastasis (28, 29). In addition, the sample size of biopsy tissues is small which can easily leading to missed diagnosis (30). In addition, the biopsy is not recommended as a routine test for diagnosis of HCC by the current guidelines (6, 31). Therefore, the current preoperative detection of CK19 is somewhat limited. Radiomics is a powerful tool for modern precision medicine (32). It captures high-throughput radiomics features from medical images combined with clinically relevant information to further improve the accuracy of diagnosis and prognosis prediction, since these features can provide additional information, such as tumor phenotypes and immune microenvironment (33). As one field of radiomics, ultrasomics plays an important role in the diagnosis and treatment of liver cancer (34). Mao et al. successfully classified primary and metastatic liver cancer using k-nearest neighbor, logistic regression, multilayer perceptron, random forest, and SVM algorithms based on grayscale ultrasound images (35). Based on ultrasound original radio frequency signals of HCC, Dong et al. effectively predicted MVI using sparse representation algorithm and machine learning algorithm combined with signal analysis and processing techniques (36). Ma et al. developed a radiomics model based on dynamic contrast-enhanced ultrasound (CEUS) (37). They found that the model performed well in predicting early HCC recurrence after ablation, while combining CEUS, US radiomics and clinical Combination models of factors can stratify high risk of late recurrence. The above studies demonstrate that multiple modalities of ultrasomics can successfully predict diagnosis and differential diagnosis of HCC, early recurrence and key prognostic factors. At present, some studies have successfully constructed radiomics models for predicting CK19 status based on Magnetic resonance imaging (MRI) images with good performance (38–40). However, MRI cannot be applied to some special populations, such as those with claustrophobia or metal-containing implants in their bodies. Furthermore, MRI is time-consuming and expensive, which limits its clinical application (41). Ultrasound has become one of the most common examination methods for the liver because of its non-invasive and non-radiative properties, more applicable population, repeated observation and relatively low cost (42). As a branch of radiomics, ultrasomics has been successfully applied to the accurate diagnosis of various malignant tumors, such as liver cancer, thyroid cancer and breast cancer, with good results (43–47). However, there are few reports about the prediction of CK19 expression in HCC patients based on ultrasomics method.

Therefore, this study aims to explore the value of machine learning-based ultrasomics for non-invasive prediction of CK19 expression in HCC patients, and to further evaluate the generalization ability of the prediction model using an independent external validation dataset.

## Materials and methods

### Study population

**Figure 1:**
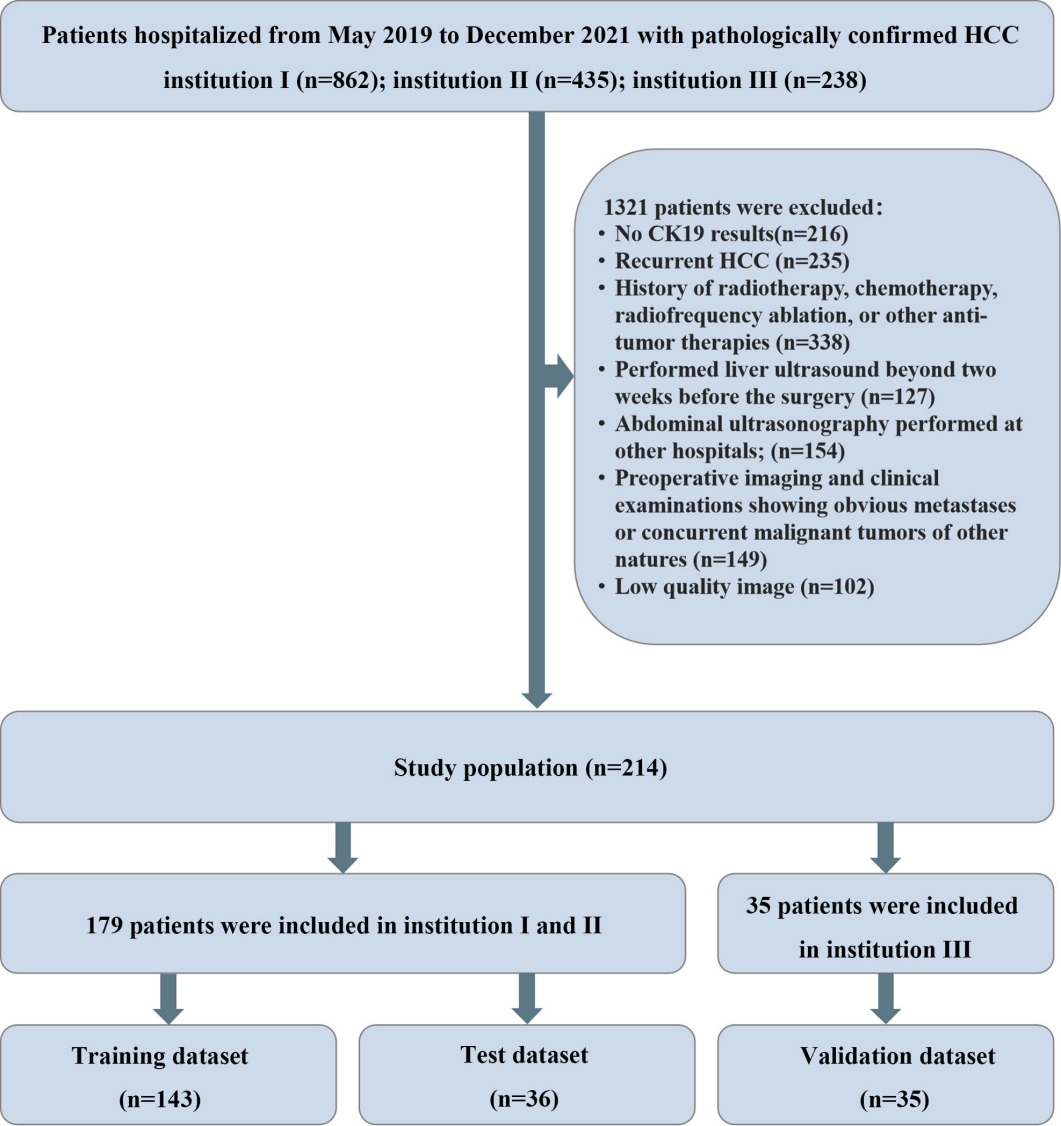
Flowchart: Cases were screened and enrolled according to the established exclusion criteria.

This retrospective study was approved by the ethics review boards of three medical institutions, Henan Provincial People’s Hospital (Institution I), the First Affiliated Hospital of Zhengzhou University (Institution II), and Henan Cancer Hospital (Institution III), and the patients’ informed consent was waived. From May 2019 to December 2021, clinical, pathological and imaging data of 1535 hospitalized patients from the above three medical institutions were collected, and the population was screened according to the following criteria. Inclusion criteria: (1) pathologically confirmed HCC with CK19 results; (2) performed liver ultrasound with two weeks before the surgery; (3) clinical and imaging data integrity. Exclusion criteria: (1) recurrent HCC; (2) history of radiotherapy, chemotherapy, radiofrequency ablation, or other anti-tumor therapies; (3) abdominal ultrasonography performed at other hospitals; (4) preoperative imaging and clinical examinations showing obvious metastases or concurrent malignant tumors of other natures; (5) low quality image. A total of 214 patients were finally included in this study, of which 179 patients from institution I and II were divided into training dataset (n = 143) and test dataset (n = 36) by random stratified sampling (ratio, 8:2), and 35 patients from institution III served as an independent external validation dataset. The screening and grouping flow chart of the study population is shown in **Figure 1**.

The indicators of the patients mainly included gender, age, hepatitis B surface antigen (HbsAg)/hepatitis C virus antibody (HCV-Ab), serum liver and kidney function indicators, coagulation function indicators, liver cirrhosis, splenomegaly, tumor location, maximum tumor diameter and tumor number. HCC specimens from all patients were pathologically examined and diagnosed according to World Health Organization criteria. In this study, all patients were divided into CK19-positive and CK19-negative groups, where CK19 positive is defined as the presence of membranous or cytoplasmic immunoreactivity in ≥5% of tumor cells (21).

### Image acquisition, preprocessing and ROI segmentation

All patients fasted for more than 8 hours before abdominal ultrasonography. Preoperative ultrasonography was performed by sonographers with more than 10 years of experience in liver ultrasonography, and the echogenicity, lesion size, and blood flow signals of the lesions were also assessed and recorded. At least one original ultrasound image clearly showing the maximum lesion diameter and one original ultrasound image containing the measured parameters in the same section should be stored in Digital Imaging and Medicine Communication (DICOM) format, respectively. The models of ultrasound equipment used were: GE Logiq E20, GE Vivid E9, HIVISION Ascendus, HIALOK ProSound A5, Philips EPIQ 7 or Philips EPIQ 5, etc. All ultrasound probes were C75, with the frequency of 1 – 5 MHZ.

In order to eliminate differences caused by different ultrasound equipment and different operators and to ensure the comparability of the features, researchers with 6 years of experience carried out image preprocessing. To ensure the distribution of baseline features, we first used stratified sampling to divide the training dataset and test dataset for patients in institutions I and II in a ratio of 8:2. Then, we used b-spline for ultrasound images reconstructed with different voxel sizes. The images were resampled to a pixel size of 1 mm x1 mm, and gray-level discretized in the histogram with the bin width set to a fixed 25.

**Figure 2:**
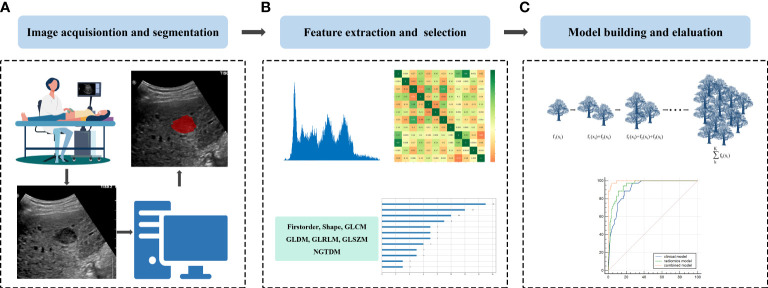
Schematic diagram of the overall study: **(A)** Image acquisition and lesion segmentation; **(B)** Feature extraction and feature selection, and **(C)** Model construction and evaluation.

**Figure 3:**
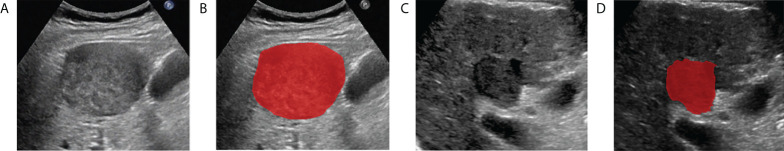
Examples of delineating regions of interest (ROI) on a grayscale ultrasound image. **(A, B)** are the CK19-positive HCC patient, **(C, D)** are the CK19-negative HCC patient.

Region of interest (ROI) segmentation for this study was performed by a sonographer with 30 years of experience in abdominal ultrasound diagnosis (sonographer 1), and a sonographer with 10 years of experience in abdominal ultrasound diagnosis (sonographer 2). First, under the guidance of sonographer 1, sonographer 2 used ITK-SNAP software (http://www.itksnap.org) to manually segment each patient’s ultrasound image along the lesion margin on the largest transverse section of the tumor. To assess the reproducibility of features, 50 cases of the ultrasound images were randomly selected for segmentation by the sonographer 2. Both sonographers were blinded to the clinical and pathological data of all patients. The flowchart of this research is shown in **Figure 2**. The representative lesion segmentation images are shown in **Figure 3**.

### Ultrasomics feature extraction and screening

First, 14 filters were used to process the original image of each patient to obtain the corresponding derived images, and then the open-source software package pyradiomics 2.1.2 was used to extract the information in all original images and derived images in high-throughput and converted them into quantitative features. Seven major radiomics features below were obtained: first order, shape, gray level co-occurrence matrix (GLCM), gray level run length matrix (GLRLM), gray level size zone matrix (GLSZM), neighboring gray tone difference matrix (NGTDM) and gray level dependence matrix (GLDM). After extracting all feature values, the missing value of each feature was filled with the median. Finally, the data were normalized (Z-score normalization) according to the mean and standard deviation to make the data conformed to a normal distribution. Details of the feature extraction methods and the filters used are provided in the **Supplementary Material 1**.

The optimal feature subset was selected by feature dimensionality reduction as follows: firstly, the reproducibility of the extracted features was evaluated by the intraclass correlation coefficient (ICC), where the features with ICC > 0.8 were considered to be reproducible (48); secondly, the features with variance of 0 (i.e., features that did not contribute anything to the classification) were excluded by the variance threshold; thirdly, the linear or nonlinear information relationships between each feature and the label were captured by mutual information, and the features with maximal information coefficient (MIC) of 0 were filtered. Finally, dimensionality reduction was further performed using the embedding method in combination with eXtreme Gradient Boosting (XG Boost).

### Model construction and evaluation

The ultrasomics model, the clinical model and the combined model were constructed using the eXtreme Gradient Boosting (XGBoost) algorithm in combination with the learning curve and the grid search for tuning parameter, respectively. XGBoost, an efficient and widely used machine learning algorithm, incorporated regularization and parallel processing, which could reduce both overfitting and computation (49).

Firstly, the ultrasomics model was constructed using the optimal ultrasomics signatures selected above. Secondly, the clinical model was constructed by 21 clinical features, including gender, age, Child-Pugh classification, HbsAg/HBC Ab (positive/negative), cirrhosis (yes/no), splenomegaly (yes/no), tumor location, tumor maximum diameter, tumor number, and serum biochemical parameters, including alpha-fetoprotein (AFP), alanine aminotransferase (ALT), aspartate aminotransferase (AST), alkaline phosphatase (ALP), glutamyl-transpeptidase (GGT), albumin, total bilirubin (TB), conjugated bilirubin (CB), creatinine, prothrombin time (PT), fibrinogen, international normalized ratio (INR). Finally, the combined model was constructed based on all the above clinical features and ultrasomics signatures to explore whether the combination of the two can show better performance. **Supplementary Material 2** includes details of parameter tuning for model building.

The performance of the three prediction models was assessed in the test dataset and the external validation dataset, and expressed as four indicators: area under the receiver operating characteristic curve (AUC) with 95% confidence interval (CI), accuracy, sensitivity and specificity. Model construction and evaluation were performed in the Python environment using the scikit-learn 0.23.2 package.

### Statistical analysis

Statistical analysis was performed by IBM SPSS Statistics 23.0 software. The distribution of continuous variables was first determined by the Shapiro – Wilk test, expressed as mean ± standard deviation or median (25th to 75th percentile) for continuous variables. Categorical variables were expressed as frequency and relative frequency. Statistical differences between the two groups of CK19-positive and CK19-negative patients were then analyzed as described above using t-test or Mann-Whitney U test for continuous variables and chi-square test or Fisher’s exact probability test for categorical variables. A value of *p* < 0.05 was considered statistically significant.

## Results

### Baseline characteristics of the study population

A total of 214 HCC patients were finally included in this study. Patients from institution I and II were mixed and divided into training dataset (n = 143) and test dataset (n = 36) by random stratified sampling (ratio, 8:2), and patients from institution III separately served as external validation dataset (n = 35). In the whole study cohort, CK19 negative and positive patients accounted for 78.97% (169/214) and 21.03% (45/214), respectively, and male and female patients accounted for 80.37% (172/214) and 19.63% (42/214), respectively. The baseline clinical and pathological characteristics of all patients are shown in **Table 1**.

**Table 1:** Preoperative clinical baseline characteristics of 214 patients.

Clinical characteristics	CK19- (n = 169), n (%)	CK19+ (n = 45), n (%)	*p* value
Sex			0.181
male	139 (82.25%)	33 (73.33%)	
female	30 (17.75%)	12 (26.67%)	
Age (years)	56.30 ± 11.079	55.16 ± 10.388	0.535
Child-Pugh Class			0.041
A	151 (89.35%)	35 (77.78%)	
B	18 (10.65%)	10 (22.22%)	
HbsAg/HCV Ab			0.980
positive	128 (75.74%)	34 (75.56%)	
negative	41 (24.26%)	11 (24.44%)	
Cirrhosis			0.922
Yes	140 (82.84%)	37 (82.22%)	
No	29 (17.16%)	8 (17.78%)	
Splenomegaly			0.391
Yes	78 (46.15%)	24 (53.33%)	
No	91 (53.85%)	21 (46.67%)	
AFP (ng/ml)	14.60 (4.79-280.84)	33.80 (5.64-589.68)	<0.001
ALT (U/L)	29.00 (20.15-46.9)	31.00 (21.00-48.00)	0.274
AST (U/L)	86.00 (69.00-114.00)	36.00 (25.00-49.85)	0.831
ALP (U/L)	86.00 (69.00-114.00)	88.50 (69.00-114.08)	0.283
GGT (U/L)	54.00 (30.15-103.50)	54.00 (30.00-113.50)	0.384
Albumin (g/L)	40.80 (36.90-44.40)	40.80 (36.98-44.40)	0.752
TB (umol/L)	13.2 (9.50-18.70)	13.70 (9.65-19.88)	0.070
CB (umol/L)	5.20 (3.50-7.80)	5.25 (3.70-7.80)	0.216
Creatinine (umol/L)	65.00 (56.00-76.00)	64.00 (56.00-75.25)	0.222
PT (s)	12.30 (11.40-13.20)	12.30 (11.40-13.20)	0.924
Fibrinogen (g/L)	2.44 (1.95-2.88)	2.45 (2.00-2.92)	0.108
INR	1.04 (0.98-1.11)	1.05 (0.98-1.11)	0.952
Tumor location			0.729
right lobe	139 (82.25%)	38 (84.44%)	
left lobe	30 (17.75%)	7 (15.56%)	
Maximum diameter (mm)	42.00 (28.00-67.00)	41.00 (27.00-66.25)	0.203
Tumor Number			0.208
1	136 (80.47%)	33 (73.33%)	
2	12 (7.10%)	7 (15.56%)	
>2	21 (12.43%)	5 (11.11%)	

CK19, Cytokeratin 19; AFP, alpha-fetoprotein; ALT, alanine aminotransferase; AST, aspartate aminotransferase; ALP, alkaline phosphatase; GGT, glutamyl-transpeptidase; TB, total bilirubin; CB, conjugated bilirubin; PT, prothrombin time; INR, international normalized ratio; Unless otherwise specified, data in parentheses are percentages.

### Feature extraction and screening

A total of 1,409 features were extracted from the original and derived images, including first order, shape, GLCM, GLRLM, GLSZM, NGTDM and GLDM of 18, 14, 24, 16, 16, 5 and 14, respectively. All but 14 shape features were obtained from the original and derived images. Details of the features were provided in the **Supplementary Material 3**.

Firstly, a total of 992 features were retained according to the ICC of features. Secondly, 16 features with zero variance and 487 features with zero MIC were excluded using variance threshold and mutual information. Finally, 12 most valuable signatures were selected using the embedding method combined with XGBoost for further dimension reduction. **Supplementary Figures 1, 2** showed the importance of the 12 signatures.

### The performance Of ultrasomics, clinical and combined models

**Figure 4:**
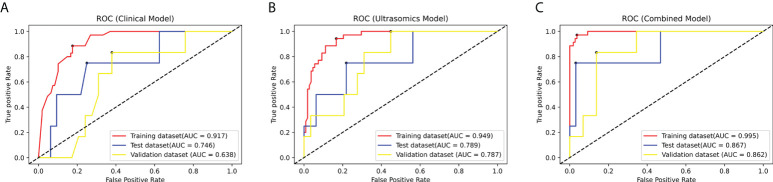
The ROC curves of the modes in the training dataset, test dataset and validation dataset: **(A)** The clinical model. **(B)** The ultrasomics model. **(C)** The combined model.

Three prediction models, that’s ultrasomics model, clinical model and combined model, were constructed using XGBoost algorithm, respectively. The results showed that the ultrasomics signatures showed satisfactory performance in predicting CK19 expression in HCC patients, and the AUCs of the test dataset and the external validation dataset were 0.789 (95% CI, 0.621 – 0.907) and 0.787 (95% CI, 0.616 – 0.907), respectively. The AUC of the clinical model constructed based on the relevant clinical characteristics was 0.746 (95% CI, 0.574 – 0.876) and 0.638 (95% CI, 0.459 – 0.793) in the test and validation datasets, respectively. However, when the clinical features and ultrasomics features were combined, the combined model achieved an excellent performance in predicting CK19 expression, and the AUC increased to 0.867 (95% CI, 0.712 – 0.957) and 0.862 (95% CI, 0.703 – 0.955), respectively. The ROC curves of all models in the three datasets are presented in **Figure 4**, and the detailed indicators of performance evaluation are presented in **Table 2**.

**Table 2:** The performance of training dataset, test dataset and verification datase.

Dataset	Model	Accuracy (%)	Sensitivity (%)	Specificity (%)	AUC (95%CI)	*p* value
Training dataset	Clinical	82.52	88.57	80.56	0.917 (0.859-0.956)	<0.0001
	Ultrasomics	85.31	88.57	84.26	0.949 (0.899-0.979)	<0.0001
	Combined	95.80	94.29	96.30	0.995 (0.965-1.000)	<0.0001
Test dataset	Clinical	63.89	75.00	62.50	0.746 (0.574-0.876)	0.0750
	Ultrasomics	77.78	75.00	78.12	0.789 (0.621-0.907)	0.0289
	Combined	86.11	75.00	87.50	0.867 (0.712-0.957)	0.0016
Validation dataset	Clinical	62.86	83.33	58.62	0.639 (0.459-0.793)	0.2513
	Ultrasomics	71.43	66.67	72.41	0.787 (0.616-0.907)	0.0011
	Combined	85.71	83.33	86.21	0.862 (0.703-0.955)	<0.0001

AUC, area under the receiver operating characteristic curve; CI, confidence interval.

## Discussion

HCC with positive CK19 expression is a new subtype of primary liver cancer (16, 25). In HCC, positive CK19 expression is one of the independent risk factors for prognosis, and is significantly correlated with invasion, chemotherapy drug resistance, and lymph node metastasis (14, 15, 18, 22). It has been reported that recurrence-free survival was significantly reduced in CK19-positive patients after surgical resection compared with CK19-negative HCC patients, with 26.1% decrease in 1-year survival, 16% decrease in 2-year survival, and 16.4% decrease in 3-year survival, which seriously affected prognosis of the patients (50). Therefore, preoperative assessment of CK19 expression in HCC patients is critical for the development of individualized treatment strategies, and improving the prognosis of patients. Preoperative immunohistochemistry is the main choice for clinical detection of CK19 (27). However, preoperative tissue biopsy may increase the risk of unwanted complications, such as abdominal or subcapsular hemorrhage, as well as needle tract metastasis (28, 29). Meanwhile, in current guidelines, biopsy is not a routine test for HCC diagnosis (6, 31). Therefore, the current preoperative detection of CK19 is somewhat limited.

Radiomics could extract a large number of macro unrecognizable, high- dimensional features through advanced data mining technology to help clinicians to further improve the accuracy of diagnosis and prognosis prediction (32). As a field of radiomics, ultrasomics plays an important role in the diagnosis and treatment of liver cancer (34). In this multicenter study, we fully mined the high-throughput information in gray-scale ultrasound images, and constructed and validated three models to predict CK19 expression in HCC patients. Firstly, we extracted a total of 1,409 ultrasomics features from the original and derived images. In order to avoid curse of dimensionality, we used ICC, variance threshold, and embedding method combined with XGBoost to reduce the dimensionality of the features, resulting in 12 optimal signatures. Then, the XGBoost algorithm combined with the learning curve and the grid search parameter adjustment method was used to train three prediction models: the ultrasound omics model, the clinical model and the combined model. The results showed that ultrasomics signatures based on machine-learning could predict and classify the expression of CK19 in HCC.As can be seen from **Table 2**, the combined model incorporating ultrasomics signatures and clinical factors performed excellently, with AUC improving to 0.867 (95% CI, 0.712 – 0.957) and 0.862 (95% CI, 0.703 – 0.955), respectively. In addition, in external validation dataset, the combined model not only reached an AUC of more than 0.85, but also reached an accuracy, sensitivity, and specificity of more than 80% (85.71%, 83.33%, and 86.21%, respectively), which indicated that the combined model had a more stable performance. Notably, the reproducibility of the results is one of the main limitations of radiomics in clinical application, but the current radiomics prediction studies of HCC are mostly based on a single center. On one hand, the heterogeneity of the images collected by the single center is relatively low, and the model had not been verified externally, which might be an overfitting phenomenon. On the other hand, the cases selected from multiple centers have a wide range of disease distribution and other aspects compared to a single center study. These were the reasons why we conducted a multicenter study. The results showed that model performance on the test dataset were comparable to the performance on the external validation dataset, especially the AUCs of the combined model were as high as 0.86. Therefore, the models had a reliable generalization ability.

To date, only a few scholars explored the correlation between HCC radiomics signatures and CK19 expression, mostly based on MRI (38–40). Wang et al. identified HCC patients with positive CK19 expression based on texture features of conventional MRI image sequences (38). They manually segmented lesions and extracted texture features in diffusion-weighted imaging (DWI) sequences, and then analyzed 7 conventional sequence MRI appearances, clinicopathological characteristics, and 2,415 texture features using univariate and multivariate analysis methods. Finally, serum AFP level ≥ 400 ng/mg, arterial rim enhancement, and StdSeparation 3D texture features were identified as predictive variables associated with CK19 positivity in HCC patients, and then a logistic regression prediction model was constructed using the above variables. The AUCs predicted by each of the three factors was 0.650 (95% CI, 0.533 – 0.754), 0.635 (95% CI, 0.518 – 0.741), and 0.765 (95% CI, 0.655 – 0.853), respectively. While combining the three characteristics, the prediction model performed optimally, with an AUC of 0.844 (95% CI, 0.744 – 0.916). Wang et al. developed a nomogram for the prediction of CK19 expression, which incorporates both clinico-radiological features and fused radiomics features (39). They extracted 647 radiomics features from enhanced MRI multi-sequence images based on a machine learning algorithm, and then used the least absolute shrinkage and selection operator regression and decision tree for feature screening and model construction. Finally, in the validation dataset, the AUC of the radiomics model fused with 17 optimal signatures was 0.822 (95% CI, 0.716 – 0.928), and the AUC of the combined model incorporating clinical factors, conventional imaging features, and radiomics signatures reached 0.846 (95% CI, 0.730 – 0.963). Yang et al. developed four CK19 expression classifiers based on HCC-enhanced MRI images from three centers and compared their performance (40). They constructed predictive classifiers using four machine learning algorithms: multiple logistic regression, support vector machine, random forest, and artificial neural network algorithm (ANN), respectively, and evaluated the generality of the optimal classifier in two validation datasets. The results showed that the ANN classifier constructed from the 12 optimal features exhibited the best diagnostic performance. The AUC was 0.857, 0.726, and 0.790 in the training, validation 1, and validation 2 datasets, respectively. In this study, the AUC of the ultrasomics model and the combined model reached 0.789 (95% CI, 0.621 – 0.907) and 0.867 (95% CI, 0.712 – 0.957) in the test dataset, respectively. In addition, the two models also achieved similar performance in the external validation dataset, with AUC of 0.787 (95% CI, 0.616 – 0.907) and 0.862 (95% CI, 0.703 – 0.955), respectively. The results showed that although the gray-scale ultrasound images used in this study were not as rich as the image sequences contained in MRI, our ultrasomics model achieved similar prediction performance with the prediction model constructed by integrating multiple sequence radiomics features of MRI. This fully demonstrated that gray-scale ultrasound images included a variety of information and also had a great potential in predicting the level of tumor heterogeneity. In addition, our prediction model also showed excellent prediction performance in the independent external validation dataset, and the ultrasonography is relatively cheap, which makes the ultrasomics method a better choice for popularization.

However, this study also had some limitations. Firstly, this was a retrospective study and there might be selection bias. And the data came from three medical institutions, especially with relatively few positive samples. In the future, we hope to expand the research scope and increase the sample size. Secondly, the images used in this study were acquired by multiple ultrasound devices. Although feature extraction was preprocessed before, there might still be some device-related differences which were not eliminated. Again, this study extracted features from the largest section of the tumor only, and will include more sections in the future for in-depth study. Finally, only gray-scale ultrasound images were collected, but we hope to collect more ultrasound images with multiple parameters and modalities to further investigate CK19 expression prediction in HCC patients by ultrasomics.

In conclusion, ultrasomics signatures could be used for noninvasive prediction of CK19 expression in HCC, and the combined prediction of clinical features and optimal ultrasomics feature subset showed an excellent performance, which improved the prediction of CK19 expression in HCC significantly. Therefore, machine learning-based ultrasomics methods may be used to predict tumor heterogeneity and facilitate the development of precision medicine.

## Data availability statement

The original contributions presented in the study are included in the article/**Supplementary Material**, further inquiries can be directed to the corresponding author.

## Ethics statement

The studies involving human participants were reviewed and approved by 1. Medical Ethics Committee of Henan Provincial People’s Hospital (Affiliated to Henan Provincial People’s Hospital) 2. Ethics Review Committee of Scientific Research Projects of the First Affiliated Hospital of Zhengzhou University (Affiliated to the First Affiliated Hospital of Zhengzhou University) 3. Medical Ethics Committee of Henan Provincial Cancer Hospital (Affiliated to Henan Provincial Cancer Hospital. The ethics committee waived the requirement of written informed consent for participation. Written informed consent was not obtained from the individual(s) for the publication of any potentially identifiable images or data included in this article.

## Author contributions

LLZ: data collection, study design, statistical analysis and interpretation, manuscript writing. QQ, QL, XL, YW, LY: data collection and management. SR: study design and manuscript review. SL, BM: data analysis, software processing. LL, YL: study design support. SD: data collection, study design, manuscript review and funding. LZZ: manuscript review and editing, study design and supervision, project management, and funding. All authors contributed to the article and approved the submitted version.

## Funding

This study was sponsored by the National Key Research and Development Program of China (Grant No. 2018YFC0114606) and Henan Nature Science Foundation (Grant No. 212300410389), and Medical Science and Technology Breakthrough Plan Project of Henan Province (Grant No. LHGJ20210020).

## Acknowledgments

I am appreciative to LZZ and the Henan Engineering Technology Research Center of Ultrasonic Molecular Imaging and Nanotechnology s for providing the assistance and facilities to accomplish the whole research. I also want to express my thanks to Long Yang, Ye Zhang, Shuaiyang Wang, Guoxin Deng, Simeng Wang, Xiaoxia Xu and Qiwei Cheng for their assistance in data collection.

## Conflict of interest

The authors declare that the research was conducted in the absence of any commercial or financial relationships that could be construed as a potential conflict of interest.

## Publisher’s note

All claims expressed in this article are solely those of the authors and do not necessarily represent those of their affiliated organizations, or those of the publisher, the editors and the reviewers. Any product that may be evaluated in this article, or claim that may be made by its manufacturer, is not guaranteed or endorsed by the publisher.

## Abbreviations

HCC, hepatocellular carcinoma; CK19, Cytokeratin 19; MRI, Magnetic resonance imaging; MRI, magnetic resonance imaging; HBsAg, hepatitis B surface antigen; HCV Ab, hepatitis C virus antibody; AFP, alpha-fetoprotein; DICOM, digital imaging and communications in medicine; ROI, region of interest; GLCM, gray-level cooccurrence matrix; GLSZM, gray-level size-zone matrix; GLRLM, gray-level run length matrix; GLDM, gray-level dependence matrix; NGTDM, neighboring gray-tone-difference matrix; ICC intra-class correlation coefficient; ALT, alanine aminotransferase; AST, aspartate aminotransferase; ALP, alkaline phosphatase; GGT, glutamyl-transpeptidase; TB, total bilirubin; CB, conjugated bilirubin; PT, prothrombin time; INR, international normalized ratio, AUC, area under the receiver operating characteristic curve; CI, confidence interval.
