# Supplementary material for: Ultrasomics prediction for cytokeratin 19 expression in hepatocellular carcinoma: A multicenter study
Source: Front Oncol. 2022 Sep 2;12:994456. doi: 10.3389/fonc.2022.994456 (PMC9478580; doi:10.3389/fonc.2022.994456)
Supplement: Supplementary file 1 [file DataSheet_1.docx]

Supplementary Material

# Supplementary 1: Feature extraction

In this study, all radiomics features were extracted from 1 original image and 14 derived images using the Pyrodiomics v.2.1.2 software package. Derived images mainly include: Wavelet (wavelet-LLH, wavelet-LHL, wavelet-LHH, wavelet-HLH, wavelet-HLL, wavelet-LLL, wavelet-HHL, wavelet-HHH), Square, Squareroot, Logarithm, Exponential, Gradient, LocalBinaryPattern 2D.

1. **Supplementary 2: Model training**

XGBoost machine learning algorithm was used to train clinical model, ultrasomics model and combined model respectively in training dataset. First, use the learning curve to adjust the hyperparameters n_estimators, max_depth, gamma, scale_pos_weight respectively. Then use the grid search method to adjust parameters of colsample_bytree, subsample, reg_lambda, and reg_alpha.

# Supplementary 3:Feature selection

Intra-group correlation coefficient (ICC) screening, variance threshold, mutual information and embedded method combined with eXtreme Gradient Boosting (XGBoost) were successively used for feature screening. Finally, 12 ultrasomics signatures were selected for model construction, as shown below:

| No. | Feature Name | Feature importance |
| --- | --- | --- |
| 1 | wavelet-LHL_glszm_ZonePercentage | 15 |
| 2 | wavelet-LLL_firstorder_Minimum | 12 |
| 3 | wavelet-LHL_glrlm_LongRunLowGrayLevelEmphasis | 10 |
| 4 | wavelet-HHL_glcm_Autocorrelation | 9 |
| 5 | wavelet-HLH_gldm_SmallDependenceEmphasis | 7 |
| 6 | wavelet-HHL_glszm_ZonePercentage | 7 |
| 7 | squareroot_firstorder_MeanAbsoluteDeviation | 7 |
| 8 | square_glrlm_RunVariance | 6 |
| 9 | wavelet-HLH_glszm_SmallAreaHighGrayLevelEmphasis | 5 |
| 10 | wavelet-HHH_glcm_SumSquares | 5 |
| 11 | original_firstorder_Median | 3 |
| 12 | square_firstorder_Mean | 3 |

1. **Supplementary Figures**


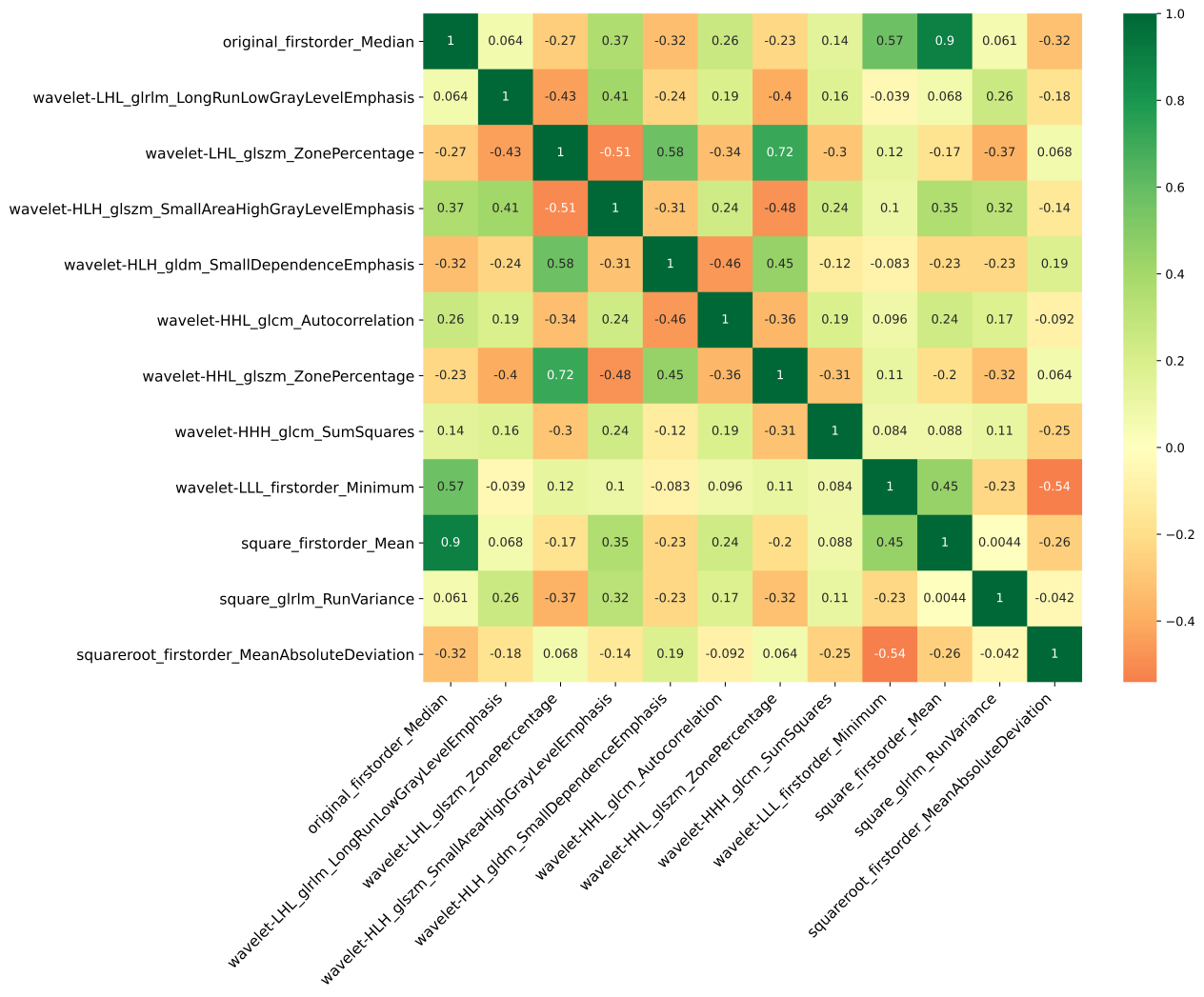


**Supplementary Figure 1.** The feature heat-map displays the correlation between 12 important radiomics signatures extracted from the training data set by color. The greener the color, the higher the correlation. Conversely, the redder the color, the lower the correlation.


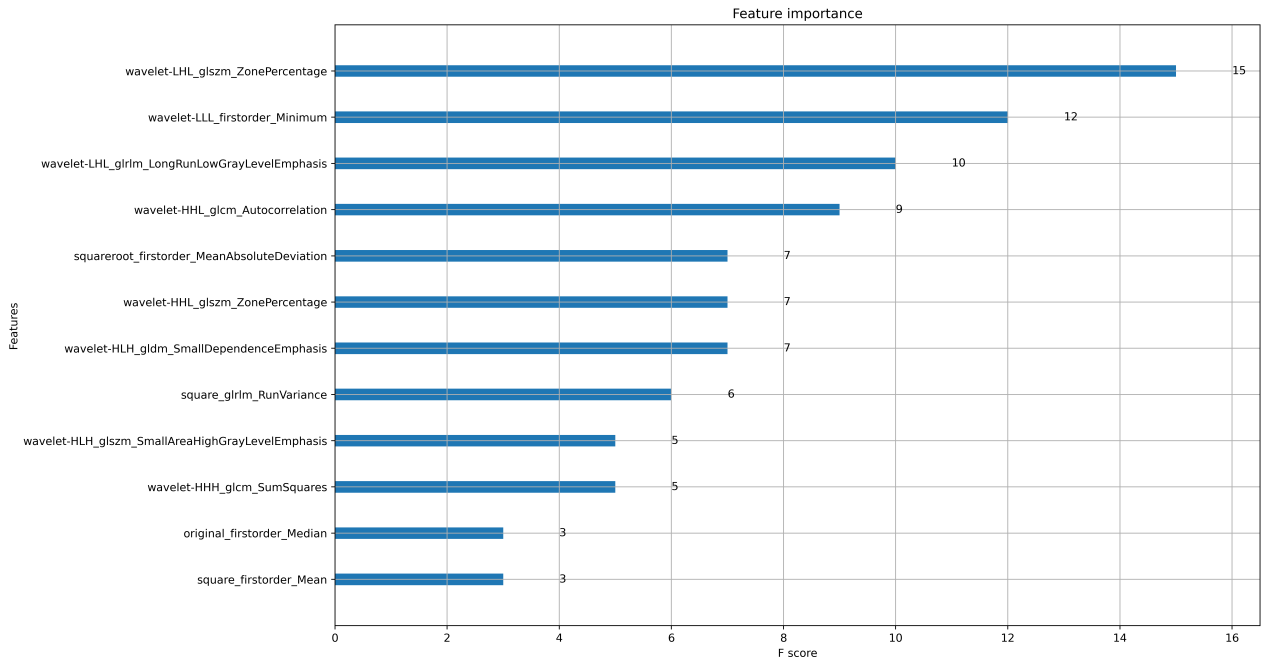


**Supplementary Figure 2.** The feature significance graph shows 12 important radiomics signatures and their corresponding feature coefficients.
